# Anaerobic Digestion of Tetracycline Spiked Livestock Manure and Poultry Litter Increased the Abundances of Antibiotic and Heavy Metal Resistance Genes

**DOI:** 10.3389/fmicb.2020.614424

**Published:** 2020-12-18

**Authors:** Getahun E. Agga, John Kasumba, John H. Loughrin, Eric D. Conte

**Affiliations:** ^1^USDA, Agricultural Research Service, Food Animal Environmental Systems Research Unit, Bowling Green, KY, United States; ^2^Department of Chemistry, Western Kentucky University, Bowling Green, KY, United States

**Keywords:** antimicrobial resistance, antimicrobial resistance genes, anaerobic digestion, tetracycline resistance, heavy metal resistance, animal manure, poultry litter, bacteria

## Abstract

Anaerobic digestion is used for the treatment of animal manure by generating biogas. Heavy metals cause environmental pollutions and co-select for antimicrobial resistance. We evaluated the impact of mesophilic anaerobic digestion of cattle manure (CM), swine manure (SM) and poultry litter (PL) on the concentrations of seven tetracycline [*tet*(A), *tet*(B), *tet*(G), *tet*(M), *tet*(O), *tet*(Q), and *tet*(W)], macrolide [*erm*(B)], methicillin (*mec*A and *mec*C), copper (*cop*B, *pco*A, *pco*D, and *tcr*B) and zinc (*czr*C) resistance genes, and three bacterial species (*E. coli*, *Enterococcus* spp. and *Staphylococcus aureus*). The total bacterial population and total abundance of the seven *tet* genes significantly increased in the three manure types after digestion. Concentration of *tet*(M) was strongly correlated with that of *erm*(B) and enterococci. As concentration of tetracyclines declined during anaerobic digestion, that of four *tet* genes (A, B, Q, and W) and 16S rRNA increased, that of *tet*(M) decreased, and that of *tet*(G) and *tet*(O) did not change. Concentrations of *cop*B and *pco*A did not change; while that of *pco*D did not change in the PL, it increased in the SM and CM. While the concentration of enterococci remained unchanged in CM, it significantly increased in the PL and SM. Concentrations of *tcr*B significantly increased in the three manure types. While concentrations of *S. aureus* significantly increased in the CM and PL, that of SM was not affected. Concentrations of *mec*C significantly increased in all manure types after digestion; while *mec*A concentrations did not change in the SM, they significantly increased in CM and PL. While concentration of *czr*C remained low in the CM, it increased in the PL but declined in the SM. In conclusion, while mesophilic anaerobic digestion of animal manure decreased concentration of tetracyclines, it increased the concentrations of total bacteria, *tet* genes, *E. coli*, enterococci and *S. aureus* and methicillin resistance genes. It did not have any effect on concentrations of heavy metals; concentrations of heavy metal resistance genes either increased or remained unaffected depending on the animal species. This study showed the need for post-digestion treatments of animal manure to remove bacteria, antibiotic resistance genes, heavy metals and their resistance genes.

## Introduction

Antibiotics play a significant role in food animals to treat, prevent and control bacterial infections. Although the use of medically important antibiotics for growth promotion are banned in many countries, they are still used in other parts of the world (OIE, 2020). Tetracyclines are the most widely used antibiotics in food animals in the United States (FDA, 2019) and worldwide (OIE, 2020) making it a good choice to evaluate mitigation strategies to reduce antibiotic resistance determinants. Antibiotics are released into the environment through feces and urine mostly unchanged or as transformation products along with antibiotic resistant bacteria (ARB) and associated antibiotic resistance genes (ARGs) (Williams-Nguyen et al., 2016; Oliver et al., 2020). Once in the environment, antibiotics can exert selection pressure on bacteria (Pruden et al., 2006) resulting in the propagation and spread of ARB via hydrologic processes beyond the point of use, consequently resulting in environmental and public health concerns (Peak et al., 2007). Antibiotic resistant bacteria can cause severe, difficult to treat, and sometimes fatal infections, with groundwater serving as a potential source of antimicrobial resistant pathogens in the human food chain (Chee-Sanford et al., 2001; Campagnolo et al., 2002) or when animal manure is land applied as soil amendment (Miller et al., 2019). Several studies reported multiple ARGs in various environments including water, sludge, farm soils, sediment, animal manure, and municipal wastewater (Chen et al., 2010; Knapp et al., 2010; Munir and Xagoraraki, 2011; Jiang et al., 2013; Agga et al., 2015a, 2019; Rodriguez-Mozaz et al., 2015; Sui et al., 2016). Manure from livestock and poultry farming plays an important role in the dissemination of ARB and ARGs in the environment when applied as fertilizer on agricultural farms. Manure is a reservoir of ARB and antibiotic compounds, and its application on agricultural soils can significantly increase ARGs and selects for ARB populations in the farm soils and other environmental compartments (Heuer et al., 2011; Udikovic-Kolic et al., 2014; Miller et al., 2019; Meyers et al., 2020). Cattle and swine manure storage lagoons are known to carry ARGs including various tetracycline resistance (*tet*) genes (Koike et al., 2007; Peak et al., 2007).

Heavy metals such as copper (Cu) and zinc (Zn) are widely used in animal agriculture as normal nutrient requirements in the form of feed supplements. Copper and Zn are also added to animal feed in higher concentrations than required for growth promotion, disease prevention and therapy (Rensing et al., 2018). They are particularly considered as a potential alternative to antibiotics in food animals due to increased pressure to avoid the use of medically important antibiotics for growth promotion or their routine use for disease prevention. However, concerns are growing with the excessive use of heavy metals since studies have shown the use of Cu and Zn are associated with antibiotic resistance and heavy metal resistance co-selects for antibiotic resistance genes (Yazdankhah et al., 2014). Moreover, environmental pollution is a concern. They are excreted in feces and contaminate water sources and plants from animal manure runoff or from manure land application as a soil amendment and persist in the environment leading to environmental toxicity (Jensen et al., 2016; Rensing et al., 2018).

Animal manure management technologies such as composting, anaerobic digestion (AD), aerobic digestion, chemical stabilization, and others can be employed to treat animal manure to reduce the concentrations of antibiotic residues, bacteria and ARGs before disposal and land application (Zhang et al., 2015). Anaerobic digestion is a widely used manure treatment technology because of its ability to reduce the volume of the manure, remove pathogens and ARGs, and simultaneously produce useful biogas (Sahlström, 2003; Novak et al., 2007; Ma et al., 2011; Kwietniewska and Tys, 2014). Anaerobic digestion of swine lagoon (Sui et al., 2016), and municipal wastewater solids (Diehl and LaPara, 2010) resulted up to 1.34 logs reductions in the concentrations of tetracycline, sulfonamide and macrolide resistance genes. However, our recent study found that depending on the amount and frequency of addition of feed to the digesters, AD of swine manure either reduced, increased, or had no significant effect on the abundances of the *tet* genes quantified (Couch et al., 2019). Similarly, Chen et al. reported that AD and lagoon storage did not reduce the abundances of macrolide (*erm*) and *tet* genes in swine manure (Chen et al., 2010).

Because of the discrepancies in the previous studies regarding the effect of AD on ARGs in animal manure, more research on AD as an on-farm manure treatment technology is still desired. In a study (Kasumba et al., 2019) that evaluated the effect of mesophilic AD of cattle and swine manure, and poultry litter on the concentrations of tetracyclines, we observed differences in the concentrations of Cu and Zn by animal species. The objectives of this study were to evaluate the effect of AD of livestock and poultry manure on the abundances of seven *tet* genes *tet*(A), *tet*(B), *tet*(G), *tet*(M), *tet*(O), *tet*(Q), and *tet*(W), heavy metal (Cu and Zn) resistance genes, and macrolide resistance gene *erm*(B) and three bacterial pathogens *E. coli*, *Enterococcus* spp., and *Staphylococcus aureus* reported to be associated with heavy metal resistance. While *tet*(A), *tet*(B), and *tet*(G) encode for efflux proteins, *tet*(M), *tet*(O), *tet*(Q), and *tet*(W) encode for ribosomal protection proteins (Roberts and Schwarz, 2016). These genes were commonly reported from swine feces (Agga et al., 2015b) and swine waste lagoons (Koike et al., 2007; Agga et al., 2015a).

## Materials and Methods

### Anaerobic Digestion Experiments

The experimental setup of the AD is previously described (Kasumba et al., 2019). Briefly, cattle manure (CM), swine manure (SM) slurry, and poultry litter (PL) were obtained from independently owned commercial farms in central Kentucky. Cattle manure was obtained from an animal kept in a pen of animals with no antibiotics at Western Kentucky University’s feedlot cattle operation. Attempts were not successful to obtain antibiotic use information from the swine and poultry farms. However, we previously reported that the corn used at the swine farm was antibiotics free which may suggest raised without antibiotics production system (Couch et al., 2019). Two 100 mL samples of SM (∼103 g each) were weighed into two separate beakers, each diluted five times with deionized (DI) water to 500 mL to approximately 5% total solids. Because CM and PL had lower moisture contents than SM, ∼50 g of CM and PL were weighed into beakers and diluted with DI water to 500 mL. Each slurry sample was spiked with a mixture of TC, CTC, and OTC adjusted to a final concentration of 1 μg/mL each in the samples. The digestion experiment was conducted in duplicates. Diluted samples were transferred to 3 L airtight polyvinyl chloride (PVC) batch reactors where the AD experiments were conducted for 64 days. On day one, 5 mL of a 50 μg/mL glucose solution (in water) was added to each reactor as an additional energy source for the microorganisms, thereafter 1 mL of the glucose solution was added every week until the end of the experiment. The headspace of each PVC reactor was blown with nitrogen gas to remove air before the AD experiment was initiated. After reactors were agitated to ensure homogeneity, 20–25 mL liquid samples were collected from each reactor every 8 days.

### DNA Extraction and Gene Quantification

Total community DNA was extracted from 500 μL of the liquid samples using the FastDNA Spin kit for soils (MP Biomedical, Santa Ana, CA, United States) following the manufacturer’s instructions. Real time quantitative PCR (qPCR) was used to quantify the concentrations of genes encoding for all bacteria (through 16S rRNA), and seven *tet* genes *tet(*A), *tet*(B), *tet*(G), *tet*(M), *tet*(O), *tet*(Q), and *tet*(W) using published primers, probes and protocols (Supplementary Table 1). The primers were obtained from Sigma-Genosys (The Woodlands, TX, United States), and the dual-labeled black hole quencher probes for the 16S rRNA TaqMan assay were from Biosearch Technologies, Inc., (Petaluma, CA, United States). The qPCR assays were performed in QuantiTect SYBR green master mix (Qiagen, Valencia, CA, United States) in a total reaction volume of 25 μL. The assay consisted of 12.5 μL, 1.5 μL of 10 μm each of the forward and reverse primers, 200 nm of probe (for 16S rRNA), and 5 μL of 10 ng of sample DNA or the standard (ranging from 10^2^ to 10^8^ copies), and 4.5 μL of water. Sample DNA was diluted to 1:500 ratio to reduce the effect of PCR inhibitors in the samples. Typical qPCR reaction consisted of initial activation at 95°C for 15 min followed by 40 cycles of denaturation at 95°C for 15 s and annealing at specific temperatures (see Supplementary Table 1) for 20 s, followed by a final extension at 72°C for 30 s. Melt curve analysis was conducted between 65°C and 95°C with an increment of 0.2°C for 1 s. qPCR reactions were run on the CFX 96 real-time PCR detection system (Bio Rad Laboratories Inc., Hercules, CA, United States). From day 0 (undigested raw manure) and day 64 (digested manure) samples for which Cu and Zn concentrations were measured (Kasumba et al., 2019) bacteria previously reported to carry Cu and Zn resistance genes, and *erm*(B) and methicillin resistance genes (*mec*A and *mec*C) were quantified using published primers and protocols (Supplementary Table 1) using QX200 droplet digital PCR (ddPCR) system (Bio Rad Laboratories Inc.). We used TaqMan probes for *E. coli* and *Enterococcus* spp., and Eva Green assays for *S. aureus*, *erm*(B), *mec*A, and *mec*C and heavy metal resistance genes.

### Data Analysis

Gene copies of total bacteria (16S rRNA), antibiotic- and heavy metal- resistance genes were analyzed as count outcomes. The effects of sampling day (i.e., AD effect) and manure type on these outcomes were analyzed by negative binomial regression using mean as a dispersion parameter. Day 0 sampling was used as a baseline to evaluate the effect of AD. Since the proportions of non-detects (i.e., zero counts) in the PL were high for *tet*(B), *tet*(G) and *tet*(Q), only CM and SM were compared. Mean gene counts were converted to log_10_/mL and plotted over sampling days. The association between total tetracycline concentration, and 16S rRNA and the *tet* genes was evaluated using a negative binomial model, and log_10_ predicted gene copies were plotted against tetracycline concentration over time. Linear regression was used to compare the concentrations of Cu and Zn (as a continuous variable) among the manure types and between the sampling days. Manure type and sampling day were first evaluated in a univariate analysis. Full models with the sampling day and manure type, and their interaction terms were first modeled, and each model was subsequently evaluated after removing a statistically non-significant term. When univariate analyses were significant, sampling day was included in the model. Associations between the heavy metals, heavy metal resistance genes and selected bacterial concentrations were analyzed by a pairwise Pearson correlation coefficient. All statistical analyses were done in STATA 16 (StataCorp LLC, College Station, TX, United States).

## Results

### Effect of Anaerobic Digestion of Animal Manure on the Abundances of Bacteria

**FIGURE 1:**
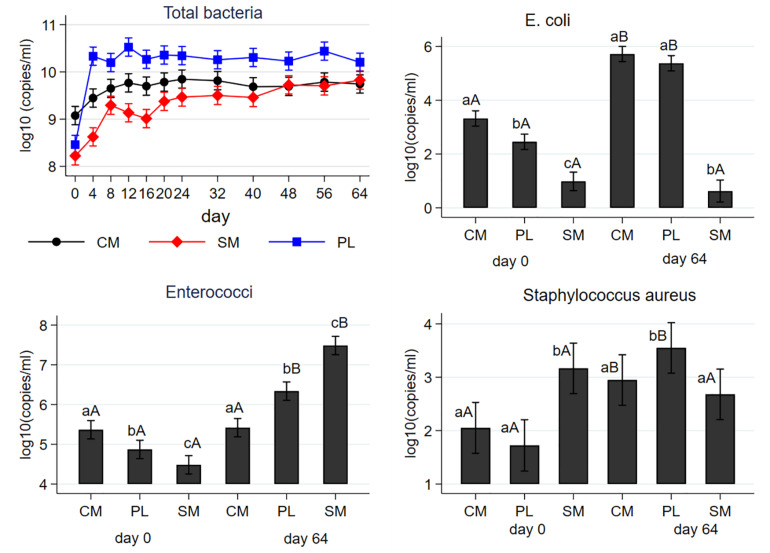
Effect of mesophilic anaerobic digestion of cattle manure (CM), swine manure (SM) and poultry litter (PL) on the mean concentrations of total bacteria (16S rRNA), *E. coli* (uidA), enterococci (23S rRNA) and *Staphylococcus aureus* (*nuc*). Data were analyzed by negative binomial regression considering gene copy numbers as count outcomes. Plots are shown as the mean concentrations along with their 95% confidence intervals. Different lower-case letters within the same sampling day indicate significant differences between the manure types; different upper-case letters within the same manure type indicate significant differences by sampling day (i.e., digestion effect). Significance was assessed at *P* < 0.05.

Mean concentrations of total bacteria, *E. coli*, enterococci, and *S. aureus* are shown in Figure 1 as a function of manure type and effect of AD. The mean concentration of the total bacteria (16S rRNA) was higher in the CM (9 logs) than SM (8.2 logs) or PL (8.5 logs) on day 0. Overall, the abundances of the total bacterial genes increased during AD in all manure types; day 64 concentrations were 4.7 (CM), 4.0 (SM), and 5.5 (PL) times higher than their respective day 0 concentrations. The 16S rRNA gene concentrations steadily increased from their baseline levels for the three manure types during the first 24 days of AD and remained unchanged thereafter. Poultry litter showed the greatest increase in the mean abundances of the 16S rRNA genes and its concentrations were higher than those of CM or SM starting on day 4 of AD. Cattle manure had the highest baseline gene copies of *E. coli* and SM had the least. While concentrations of *E. coli* significantly increased in CM and PL following AD, in SM it did not change from day 0 level. On day 64, CM and PL had similar levels of *E. coli* concentrations which was higher than that of SM. On day 0, the highest and lowest gene copies of enterococci were observed in the CM and SM, respectively. While enterococci concentrations significantly increased in both the PL and SM following AD, that of CM did not change from baseline level. On day 64, the highest and lowest concentration of enterococci were observed in the SM and CM, respectively. Concentration of *S. aureus* significantly differed both by the manure type and AD. In the pre-digested samples, the highest concentration was observed in SM with no difference between CM and PL. In the digested samples, PL had the highest concentration with no significant difference between CM and SM. While the levels of *S. aureus* significantly increased in CM and PL due to AD, that of SM did not change from its pre-digested level.

### Effect of Anaerobic Digestion of Animal Manure on the Abundances of Antibiotic Resistance Genes

**FIGURE 2:**
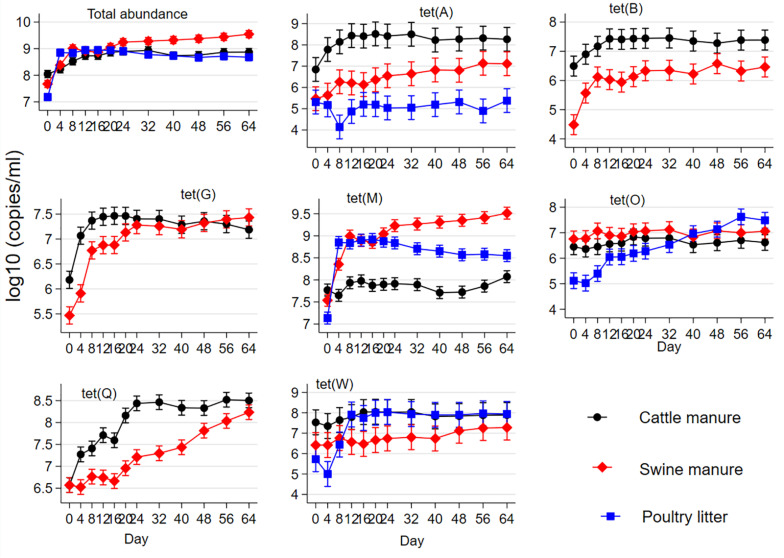
Effect of mesophilic anaerobic digestion of cattle and swine manure and poultry litter on the mean concentrations of total and individual abundances of seven tetracycline resistance genes [*tet*(A), *tet*(B), *tet*(G), *tet*(M), *tet*(O), *tet*(Q), and *tet*(W)]. Data were analyzed by negative binomial regression considering gene copy numbers as count outcomes and displayed as the mean concentrations and their 95% confidence intervals.

Combined and individual concentrations of seven tetracycline resistance genes are shown in Figure 2 as a function of manure type and sampling day. Combined total *tet* gene concentration varied at the baseline among the manure types (CM > SM > PL). Total *tet* gene concentrations increased from their pre-digestion levels in the three manure types during digestion; and total concentrations were significantly higher in the SM starting on day 24 during AD compared to CM or PL. The percentages of PL samples (n = 24) with non-detects (observations with zero gene copies i.e., with no PCR amplification) were 8.3% for *tet*(A), 83.3% for *tet*(B), 20.8% for *tet*(G) and 45.8% for *tet*(Q). Consequently, only *tet*(A) could be compared among the three manure types, while *tet*(B), *tet*(G), and *tet*(Q) were compared only between CM and SM under negative binomial models. Concentrations of *tet*(A), *tet*(B), *tet*(G), *tet*(M), *tet*(O), and *tet*(Q) increased from their baseline levels during AD in CM and SM. Concentration of *tet*(A) did not change from its baseline level in PL during AD. However, concentrations of *tet*(W) increased during AD of PL with no changes in the CM and SM. Comparing by manure type, CM had higher baseline concentrations of *tet*(A), *tet*(B), *tet*(G), *tet*(M), *tet*(O), and *tet*(W) than those of SM or PL. Concentrations of *tet*(A), *tet*(B), and *tet*(Q) in CM remained higher than that of SM or PL on most of the sampling days during AD. Concentrations of *tet*(G) in CM were higher than that of SM during the first 20 days of AD. Cattle manure had the lowest concentrations of *tet*(M) during AD compared to SM or PL.

**FIGURE 3:**
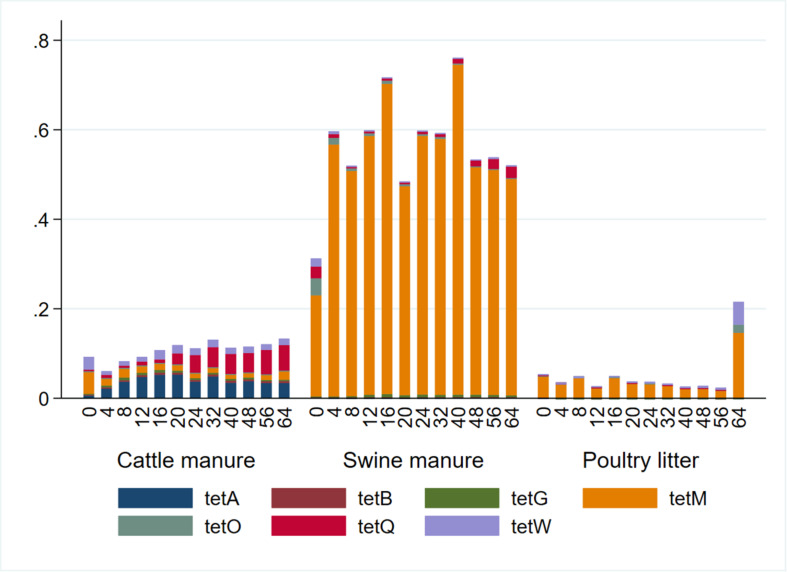
Relative abundance of seven tetracycline resistance (*tet*) genes by sampling day and manure type. Relative abundances were calculated as a ratio of the *tet* genes to 16S rRNA concentrations.

**FIGURE 4:**
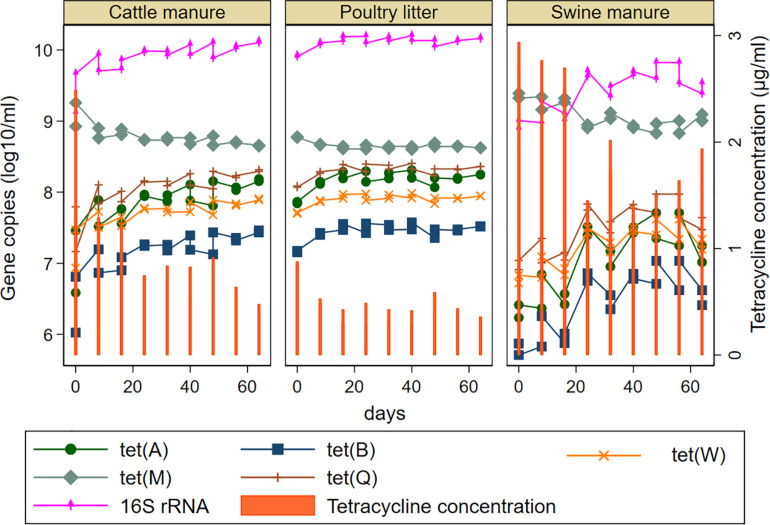
Effect of tetracycline concentrations on the model predicted abundances of total bacteria and five tetracycline resistance genes [*tet*(A), *tet*(B), *tet*(M), *tet*(Q), and *tet*(W) during mesophilic anaerobic digestion of cattle and swine manure, and poultry litter. *tet*(G) and *tet*(O) were not shown since they were not significantly associated with the concentration of tetracyclines. Predicted values for the *tet* genes was obtained after linear regression using tetracycline concentrations as a continuous variable.

Relative abundances (calculated as a ratio of *tet* gene copies per 16S rRNA gene copy) and distribution of the *tet* genes also varied considerably among the manure types (Figure 3). The abundances of the *tet* genes relative to 16S rRNA gene were considerably higher in the SM compared to CM or PL. The *tet*(M) gene had the highest relative abundances in SM and PL, while *tet*(A) and *tet*(Q) had the highest relative abundances in CM. Total tetracycline concentration generally decreased during AD regardless of the manure type (Figure 4). Total bacterial population increased as the total tetracycline concentrations decreased over time during AD in the three manure types. A similar negative association was observed for *tet*(A), *tet*(B), *tet*(Q) and *tet*(W). Interestingly, however, a positive association was observed between *tet*(M) and tetracycline concentration in which concentrations of *tet*(M) dropped as the tetracycline concentrations decreased over time regardless of manure type. *tet*(G) and *tet*(O) were not significantly associated with tetracycline concentration.

**FIGURE 5:**
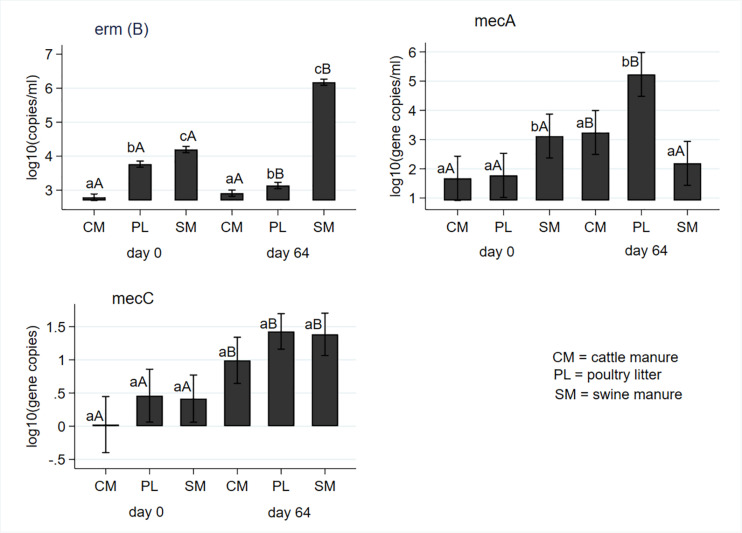
Effect of mesophilic anaerobic digestion of cattle and swine manure, and poultry litter on concentrations of macrolide *erm*(B) and methicillin (*mec*A and *mec*C) resistance genes. Data were analyzed with negative binomial regression and results were plotted as mean values with 95% confidence intervals. Different lower-case letters within the same sampling day indicate significant differences between the manure types; different upper-case letters within the same manure type indicate significant differences by sampling day (i.e., digestion effect). Significance was assessed at *P* < 0.05.

Macrolide resistance [*erm*(B)] and methicillin resistance (*mec*A and *mec*C) genes were measured at baseline (day 0) and post digestion (day 64) and results are shown in Figure 5. Concentrations of *erm*(B) significantly differed by the manure type both at the baseline and after digestion with the highest and lowest concentrations observed in the SM and CM, respectively. While concentrations in the CM remained unchanged, concentrations in the PL decreased, and concentrations in the SM significantly increased from the pre-digestion level at the end of digestion (Figure 5). Concentrations of *mec*A gene was significantly higher in the SM before digestion compared to CM or PL. However, it was significantly higher in PL than either CM or SM after digestion (Figure 5). Levels of *mec*A genes significantly increased in CM and PL with no change in SM following AD. The mean concentrations of *mec*C gene did not differ by manure type both in the pre- and post- digested samples (Figure 5). However, its concentrations significantly increased from predigested levels due to digestion in all manure types.

### Effect of Anaerobic Digestion of Animal Manure on the Concentrations of Heavy Metals, and Heavy Metal Resistance Genes

**FIGURE 6:**
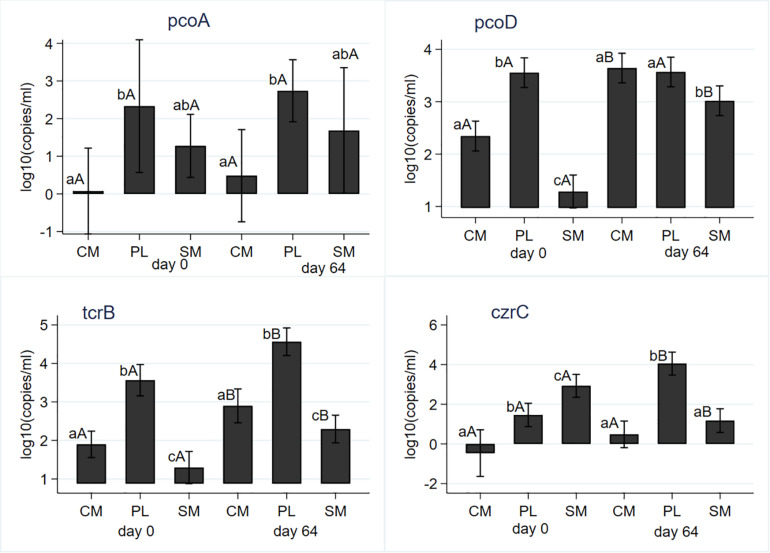
Effect of mesophilic anaerobic digestion of cattle manure (CM), swine manure (SM), and poultry litter (PL) on concentrations of copper (*pco*A, *pco*D, and *tcr*B) and zinc (*czr*C) resistance genes. Data were analyzed with negative binomial regression and results were plotted as mean values with 95% confidence intervals. Different lower-case letters within the same sampling day indicate significant differences between the manure types; different upper-case letters within the same manure type indicate significant differences by sampling day (i.e., digestion effect). Significance was assessed at *P* < 0.05.

Sampling day (i.e., AD) and its interaction with manure type did not have significant (*P* > 0.05) effects on Cu and Zn concentrations. Manure type had a significant impact on the concentrations of both heavy metals; SM had the highest concentrations of both metals and CM had the least concentrations (Table 1). Gram negative Cu resistance gene *cop*B was not detected from the PL. Its mean gene copies were significantly higher in the SM compared to CM (Table 1). Anaerobic digestion did not have any significant (*P* = 0.472) effect on the mean *cop*B copies. Mean *pco*A gene copies were not significantly (*P* = 0.608) affected by AD (Figure 6); PL had significantly higher mean gene copies than CM or SM (Table 1). The *pco*D gene differed both by AD and manure type (Table 1); undigested PL had the highest mean copies and remained unaffected by AD while mean copies in the CM and SM significantly increased during AD (Figure 6). The commonly reported transferable copper resistance *tcr*B gene in enterococci and other gram-positive bacteria was significantly higher in the PL (Table 1) both before- and after- digestion when compared to CM or SM. Mean concentrations of *tcr*B significantly increased from its pre-digestion level following digestion in all manure types (Figure 6). Zinc resistance gene *czr*C significantly differed both by the manure type and AD. The highest concentration was observed in the pre-digested SM samples; PL had the highest concentrations in the post-digestion samples. Mean *czr*C concentration significantly increased in PL, decreased in SM, and did not change in the CM (Figure 6). In general, its concentration in the CM was the lowest (Table 1).

**TABLE 1:** Comparisons of heavy metal concentrations and heavy metal resistance genes by manure type.

Heavy metal	Manure type	Mean (95% CI)			Pairwise comparisons				
		Mean	95% CI		Paired comparisons	Contrast	95% CI		*P*-value
Copper (μg/ml)	Cattle manure	0.6	0.4	0.8	PL vs. CM	12.2	6.7	17.7	**0.001**
	Poultry litter	12.8	9.0	16.7	SM vs. CM	27.3	21.8	32.8	**<0.001**
	Swine manure	27.9	24.0	31.8	SM vs. PL	15.0	9.5	20.5	**<0.001**
Zinc (μg/ml)	Cattle manure	1.8	1.3	2.3	PL vs. CM	9.4	1.8	17.1	**0.021**
	Poultry litter	11.3	5.9	16.6	SM vs. CM	33.3	25.7	41.0	**<0.001**
	Swine manure	35.2	29.8	40.5	SM vs. PL	23.9	16.3	31.5	**<0.001**
*cop*B (log_10_ copies/ml)	Cattle manure	–0.2	–0.8	0.4	PL vs. CM	N/A			
	Poultry litter	N/A			SM vs. CM	0.8	0.1	1.5	**0.033**
	Swine manure	0.6	0.2	0.9	SM vs. PL	N/A			
*pco*A (log_10_ copies/ml)	Cattle manure	0.3	–0.6	1.2	PL vs. CM	2.4	1.2	3.7	**<0.001**
	Poultry litter	2.7	1.9	3.6	SM vs. CM	1.0	–0.2	2.2	0.101
	Swine manure	1.3	0.5	2.2	SM vs. PL	–1.4	–2.6	–0.2	**0.019**
*pco*D (log_10_ copies/ml)	Cattle manure	3.4	2.9	3.9	PL vs. CM	0.2	–0.5	0.9	0.584
	Poultry litter	3.6	3.1	4.1	SM vs. CM	–0.6	–1.3	0.1	0.077
	Swine manure	2.7	2.2	3.2	SM vs. PL	–0.8	–1.5	–0.1	**0.021**
*tcr*B (log_10_ copies/ml)	Cattle manure	2.4	1.9	2.9	PL vs. CM	2.0	1.3	2.7	**<0.001**
	Poultry litter	4.4	3.9	4.9	SM vs. CM	–0.3	–1.0	0.4	0.389
	Swine manure	2.1	1.6	2.6	SM vs. PL	–2.3	–3.0	–1.6	**<0.001**
*czr*C (log_10_ copies/ml)	Cattle manure	0.2	–0.6	1.0	PL vs. CM	3.5	2.4	4.6	**<0.001**
	Poultry litter	3.8	3.0	4.5	SM vs. CM	2.4	1.3	3.5	**<0.001**
	Swine manure	2.6	1.9	3.4	SM vs. PL	–1.1	–2.2	–0.1	**<0.035**

*CM = cattle manure; PL = poultry litter; SM = swine manure; CI = confidence interval.Copper and zinc concentrations were analyzed with linear regression while gene copies of the copper resistance genes shown here were analyzed by negative binomial regression. Bonferroni adjusted pairwise comparisons of the contrast were obtained. *P*-values with bold face indicate significant difference.*

We also examined pairwise correlations between concentrations of heavy metals, heavy metal resistance genes, and three bacterial genera (*E. coli*, *Enterococcus* spp. and *S. aureus*) as shown in Table 2. Strong positive correlations were observed between *tet*(M), *erm(*B) and enterococci; between the heavy metals; between gram positive (*tcr*B) and gram-negative (*pco*A) copper resistance genes; and between methicillin- and copper- resistance genes.

**TABLE 2:** Correlation of heavy metal concentrations, heavy metal resistance genes and antibiotic resistance genes across all samples.

Variable	*erm*(B)	*tet(*M)	*mec*A	*mec*C	*cop*B	*pco*A	*pco*D	*tcr*B	*czr*C	Enterococci	*E. coli*	*Staphylococcus spp.*	Copper	Zinc
*erm*(B)	1													
*tet*(M)	0.99*	1												
	**<0.000**													
*mec*A	−0.14	−0.06	1											
	0.67	0.86												
*mec*C	0.22	0.31	0.57	1										
	0.50	0.33	0.0535											
*cop*B	0.35	0.33	−0.19	−0.26	1									
	0.26	0.30	0.56	0.41										
*pco*A	−0.15	−0.06	0.996*	**0.63**	−0.20	1								
	0.64	0.84	** <0.0001**	** 0.0289**	0.54									
*pco*D	−0.23	−0.21	−0.06	0.31	**−0.63**	−0.03	1							
	0.46	0.52	0.84	0.32	** 0.0295**	0.93								
*tcr*B	−0.15	−0.07	0.999*	**0.58**	−0.21	0.996*	−0.04	1						
	0.65	0.84	** <0.0001**	** 0.0492**	0.52	** <0.0001**	0.91							
*czr*C	−0.15	−0.11	−0.05	**0.64**	−0.19	0.04	0.50	−0.03	1					
	0.63	0.74	0.88	** 0.0257**	0.55	0.90	0.10	0.93						
Enterococci	0.996*	0.998*	−0.05	0.28	0.32	−0.06	−0.23	−0.06	−0.15	1				
	** <0.0001**	** <0.0001**	0.88	0.37	0.32	0.85	0.48	0.86	0.64					
*E. coli*	−0.27	−0.22	0.43	0.23	−0.37	0.41	0.46	0.42	−0.10	−0.23	1			
	0.39	0.50	0.17	0.48	0.23	0.19	0.14	0.18	0.75	0.48				
*Staphylococcus spp.*	−0.18	−0.10	0.84*	0.67*	−0.21	0.86*	0.01	0.83*	0.20	−0.10	0.42	1		
	0.58	0.76	**0.0007**	**0.0178**	0.51	**0.0003**	0.99	**0.0007**	0.53	0.75	0.18			
Copper	0.52	0.51	−0.05	0.25	0.45	−0.03	−0.33	−0.06	0.14	0.51	−0.49	0.20	1	
	0.08	0.09	0.87	0.44	0.15	0.93	0.29	0.86	0.67	0.09	0.10	0.53		
Zinc	0.46	0.43	−0.06	0.07	0.52	−0.06	−0.49	−0.08	−0.07	0.44	−0.44	0.18	0.96*	1
	0.13	0.16	0.84	0.83	0.09	0.86	0.10	0.81	0.84	0.15	0.15	0.57	**<0.000**	

* Shows statistically significant Pearson’s correlation coefficients. For each measured outcome, the number in the first row is a correlation coefficient and the number in the second row is the associated *P*-value. Correlation coefficients and *P*-values with statistical significance are in bold face.

## Discussion

The main goal of this study was to evaluate the impact of mesophilic AD of tetracycline-containing animal manure, on tetracycline resistance genes and the total bacterial population. We also evaluated its impact on heavy metal resistance genes, and bacterial species and ARGs associated with heavy metal resistance. The increase in the total bacterial population (16S rRNA), which could be explained by decrease in tetracycline concentration, indicates a functioning AD system perhaps predominated by strictly anaerobic bacteria (Couch et al., 2019). While anaerobic bacteria play a significant role in hydrolysis, acidogenesis and acetogenesis steps, the methanogens play a significant role in the final methanogenesis of forming methane, with hydrolysis being a rate limiting step in AD process (Appels et al., 2008). Concentrations of five of the seven *tet* genes were increased from their pre-digested levels in the manures of the three animal species; *tet*(A) increased in the CM and SM but was not affected in the PL; *tet*(W) was increased in the PL while remaining unaffected in the CM and SM (Supplementary Table 2). The dynamics (increase or not) of the *tet* genes detected could be related to specific groups of bacteria carrying them and their ability to grow under the conditions tested. Also, it could be related to the ability of the genetic elements carrying the *tet* genes to be transferred among a wide range of bacterial genera or be more restricted within some groups. Mesophilic AD of wastewater sludge under lab-scale setup, increased the concentrations of both the 16S rRNA and *tet*(G) gene and the authors concluded that mesophilic AD of municipal waste enhances the survival of ARB and horizontal gene transfer (Miller et al., 2016). Similarly, another study (Ghosh et al., 2009) reported an increase in the concentrations of tetracycline resistance genes and *int*I1, a mobilizable genetic element commonly used as an indicator of horizontal gene transfer, under mesophilic AD of municipal wastewater. In another study (Ma et al., 2011) however, mesophilic AD of municipal wastewater led to mixed results in which the concentration of *tet*(G) declined while that of *tet*(W) increased. Another study (Wallace et al., 2018) also reported that mesophilic AD of dairy manure did not have any effect on the levels of *tet*(O) and *tet*(W). The effect of mesophilic AD on the concentration of macrolide resistance gene *erm*(B) also varied by the manure type: not affected in CM, increased in SM and decreased in PL. In the mesophilic AD of wastewater study, macrolide resistance genes *erm*(B) and *erm*(F) were increased (Ma et al., 2011).

Strong negative correlations between the concentrations of tetracyclines and the 16S rRNA genes suggest that the microbial communities continued to increase during the AD process, while the tetracyclines were degraded. Similarly, the negative correlations between the tetracycline concentrations and most of the *tet* genes may in part suggest that the decrease in tetracycline concentrations potentially led to propagation of the resistant bacterial strains that increased the abundance of the *tet* genes in the manure during AD. The increase in the concentrations of four *tet* genes *tet*(A), *tet*(B), *tet*(Q), and *tet*(W) and maintenance in the other two genes *tet*(G) and *tet*(O) may indicate the persistence and propagation of tetracycline resistant bacterial population carrying these genes in the AD independent of tetracycline concentrations. While *tet*(A), *tet*(B) and *tet*(G) are exclusively detected from gram negative bacteria, *tet*(O), *tet*(Q) and *tet*(W) are found in both gram positive and gram negative bacteria (Roberts and Schwarz, 2016). Interestingly, the concentration of *tet*(M) declined as the tetracycline concentrations declined. The *tet*(M) is the most widespread gene being detected in 75 genera equally distributed between gram positive and gram-negative bacteria. This widespread distribution can be due to its association with chromosomally linked conjugative transposons with broad host range for direct transfer or linked with plasmids (Rice, 1998; Roberts and Schwarz, 2016). Drop in the concentration of *tet*(M) as the tetracycline concentration decreased could be related to its association with a variety of conjugative transposons; and conjugation modules were reported to be induced in the presence of tetracycline (Rice, 1998).

In general, variations in the concentrations of the tetracycline resistance genes in the current and previous studies suggest that microbial communities in anaerobic digesters and operating conditions that influence the development and maintenance of that community play an important role in determining the fate of ARGs (Miller et al., 2013). Furthermore, studies showed the sorption of antibiotics and antibiotic resistance genes to the biosolid following AD, which clearly indicates that mesophilic AD alone is not efficient to remove these substances and secondary treatments such as composting are required before the sludge is used as soil amendment (Wallace et al., 2018; Couch et al., 2019). On the other hand, methanogens are resistant to many of the commonly used antibiotics due to their lack of specific targets (Whitman et al., 2006).

We also quantified the effect of mesophilic AD on the concentrations of bacterial pathogens. While the concentrations of *E. coli* and *S. aureus* in the CM and PL increased following AD, they were not affected in the SM. Concentrations of *Enterococcus* spp. significantly increased in the SM and PL with no effect in the CM. Interestingly, the concentrations of *S. aureus* significantly increased in the three manure types during digestion. An increase or no effect in the concentrations of these bacterial pathogens following mesophilic AD of manure from food animals clearly indicates the need for secondary treatment of the digestate before land application. A previous study (Ziemba and Peccia, 2011) indicated that the rate of inactivation of *E. coli* and *Enterococcus faecalis* increased as the temperature of the AD increased suggesting that mesophilic digestion is not effective in removing pathogens. *S. aureus* was detected using qPCR in mesophilic and thermophilic anaerobically digested wastewater samples but not in composted mesophilic digested samples suggesting the need for post-treatment of mesophilic anaerobically digested animal manure (Viau and Peccia, 2009). A study (Börjesson et al., 2009) that was conducted in a municipal wastewater treatment plant detected *mec*A, *S. aureus* and MRSA in anaerobically digested wastewater effluent samples. Based on the detection of *S. aureus* and *mec*A in human wastewater effluent samples in these previous studies and in animal manure in the current study, it is possible to conclude that MRSA can persist in mesophilic anaerobically digested materials and that secondary treatments are required.

A strong positive correlation between copper and zinc concentrations (Table 2) can simply be due to the fact that both of them are used as feed supplements to provide essential cellular functions in both swine and poultry (Rensing et al., 2018). The effect of mesophilic AD on Cu and Zn resistance genes also varied by the manure type, much like that of the bacterial pathogens and some of the ARGs. Heavy metal resistance genes were either not affected (*cop*B and *pco*A) or increased (*tcr*B) in the three manure types (Supplementary Table 2), indicating the ineffectiveness of mesophilic AD in removing heavy metal resistance genes. Almost perfect positive correlation between concentration of enterococci, and *erm*(B) and *tet*(M) is in line with culture-based studies that reported association between macrolide resistance [*erm*(B]) and tetracycline resistance [*tet*(M)] in enterococci (Amachawadi et al., 2015). Unlike that finding, *tcr*B was not correlated with *erm*(B) and *tet*(M) in the present ecological study although these three genes have been reported to be co-located on the same genetic elements thus enabling their transfer through selection pressures from heavy metals or the antibiotics (Rensing et al., 2018). Although the positive and strong correlation between *tcr*B and *pco*A can be explained by ecological association since they were tested from same samples, it requires further studies. Both of these genes are plasmid borne and confer Cu resistance in gram positive *Enterococcus* spp. and *Enterobacteria* such as *E. coli*, respectively (Rensing et al., 2018). Another new finding in this study is the association of these two plasmid-borne Cu resistance genes and *S. aureus*, *mec*C and *mec*A. Although the use of Zn and zinc resistance gene (*czr*C) were shown to be associated with MRSA and the responsible gene (*mec*A) (Cavaco et al., 2011), an association between Cu resistance genes and MRSA has never been reported. These ecological associations generate hypotheses that need to be tested under field studies. Mesophilic AD of livestock and poultry manure did not result in significant reductions of the heavy metals Cu and Zn although these metals are known to be precipitated at pH values typical of AD (Ayres et al., 1994). The presence of heavy metal resistance genes and bacteria known to carry these genes indicates environmental and antibiotic resistance risks associated with the use of these metals in food animal production. Lack of significant effect of AD on the concentration of heavy metals can be attributed to lack of biodegradable properties of metals as supported by the detection of heavy metals after AD of municipal solid waste (Xie et al., 2015). Variations in the concentrations of the heavy metals by animal species may indicate differences in the amount of the heavy metals fed (Hejna et al., 2019), in addition to basal requirements such as for disease prevention, control and growth promotion. To mitigate these risks, a recommendation is made that concentrations of these heavy metals should be reduced and adjusted to the essential requirements in animal production (Rensing et al., 2018).

The limitation of this study was a lack of a control manure not spiked with tetracyclines. However, the effect of AD was evaluated based on before/after design comparing all outcomes to their baseline values. This method was similarly used in another study (Couch et al., 2019) by this group that evaluated the impact of AD of swine manure on the same seven tetracycline resistance genes targeted in the current study. The use of baseline values was suggested to evaluate interventions related to AMR in the absence of background values or untreated controls (Rothrock et al., 2016). In a study (Miller et al., 2013) that compared AD of sludges spiked with sulfamethoxazole and control sludge under mesophilic conditions, reductions in the concentrations of ARGs remained constant despite selection pressure. This led the authors to conclude that digester operating conditions strongly influence the bacterial community composition and ARGs compared to selective agents. Also, we do not know if the three tetracycline drugs combined and spiked into the manure samples as a mixture had any interaction effects.

## Data Availability Statement

The raw data supporting the conclusions of this article will be made available by the authors, without undue reservation.

## Author Contributions

GA, JK, JL, and EC contributed to the conception and design of the study. GA and JK performed the laboratory analysis and wrote the first draft of the manuscript. GA performed the statistical analysis. All authors contributed to manuscript revision, read and approved the submitted version.

## Conflict of Interest

The authors declare that the research was conducted in the absence of any commercial or financial relationships that could be construed as a potential conflict of interest. The handling editor declared a shared affiliation with one of the authors GA and JL at the time of review.

## Supplementary Material

Supplementary Table 1 Primer sequences used for quantification of antibiotic and heavy metal resistance and bacterial marker genes from livestock manure and poultry litter under anaerobic digestion system.

Supplementary Table 2 Summary of the effects of anaerobic digestion of cattle and swine manure, and poultry litter.
