# Supplementary material for: Anaerobic Digestion of Tetracycline Spiked Livestock Manure and Poultry Litter Increased the Abundances of Antibiotic and Heavy Metal Resistance Genes
Source: Front Microbiol. 2020 Dec 18;11:614424. doi: 10.3389/fmicb.2020.614424 (PMC7775313; doi:10.3389/fmicb.2020.614424)
Supplement: Supplementary Table 1 — Primer sequences used for quantification of antibiotic and heavy metal resistance and bacterial marker genes from livestock manure and poultry litter under anaerobic digestion system. [file Table_1.pdf]

Supplementary Table 1. Primer sequences used for quantification of antibiotic and heavy metal resistance and bacterial marker genes from livestock manure and poultry litter under anaerobic digestion system.

Target gene	Primer	Primer sequence (5'-3')[†]	Tm (°C)[‡]	size (bp)[§]	Reference
16S rRNA	16S-1055-F	ATG GCT GTC GTC AGC T	58.0	337	(Harms et al., 2003)
	16S-1392-R	ACG GGC GGT GTG TAC			
	16S-Probe-F	FAM-CAA CGA GCG CAA CCC-BHQ			
Enterococci	ECST748F	AGAAATTCCAAACGAACTTG	60.0	106	(Frahm and Obst, 2003)
	ENC854R	CAGTGCTCTACCTCCATCATT			
	ENT813probe	TGGTTCTCTCCGAAATAGCTTTAGGGCTA			
E. coli	uid784 F	GTGTGATATCTACCCGCTTCGC	60.0	82	(Frahm and Obst, 2003)
	uid866 R	AGAACGCTTTGTGGTTAATCAGGA			
	uid807 probe	TCGGCATCCGGTCAGTGGCAGT			
S. aureus	nuc-F	TCAGCAAATGCATCACAAACAG	55.0	255	(Poulsen et al., 2003)
	nuc-R	CGTAAATGCACTTGCTTCAGG			
tet(A)	tetA-F	GCT ACA TCC TGC TTG CCT TC	57.0	210	(Ng et al., 2001)
	tetA-R	CAT AGA TCG CCG TGA AGA GG			
tet(B)	tetB-F	CTC AGT ATT CCA AGC CTT TG	56.0	284	(Ng et al., 2001; Sengelov et al., 2003)
	tetB-R	GTA ATG GGC CAA TAA CAC CG			
tet(G)	tetG-F	CAG CTT TCG GAT TCT TAC GG	59.0	169	(Ng et al., 2001)
	tetG-R	CAA TGG TTG AGG CAG CTA CA			(Szczepanowski et al., 2009)
tet(M)	tetM-F	GTG CCG CCA AAT CCT TTC TG	59.0	250	(Vikram et al., 2017)
	tetM-R	GCA TCC GAA AAT CTG CTG GG			
tet(O)	tetO-F	ACG GAR AGT TTA TTG TAT ACC	58.0	170	(Aminov et al., 2001)
	tetO-R	TGG CGT ATC TAT AAT GTT GAC			
tet(Q)	tetQ-F	AGA ATC TGC TGT TTG CCA GTG	59.0	166	(Aminov et al., 2001)
	tetQ-R	CGG AGT GTC AAT GAT ATT GCA			
tet(W)	tetW-F	GAG AGC CTG CTA TAT GCC AGC	59.0	168	(Aminov et al., 2001)
	tetW-R	GGG CGT ATC CAC AAT GTT AAC			
erm(B)	ermB-F	TCACCGAACACTAGGGTTGC	60.0	131	(Vikram et al., 2017)
	ermB-R	CTGTGGTATGGCGGGTAAGT			

*mec*A	mecA-F	GGGATCATAGCGTCATTATTC	55.0	527	(Poulsen et al., 2003)
	mecA-R	AACGATTGTGACACGATAGCC			
*mec*C	mecC-F	GAAAAAAAGGCTTAGAACGCCTC	60.0	138	(Stegger et al., 2012)
	mecC-R	GAAGATCTTTTCCGTTTTCAGC			
copB	copB-F	TAGTGGCCATGCACATCATC	60.0	201	(Argudín et al., 2013)
	copB-R	CCACCAGACAAGAACGGTTT			
pcoA	pcoA-F	CGGGTATGCAAAGTCATCCT	55.0	136	(Chalmers et al., 2018)
	RT-pcoA-R	TCCGCGTACGTGAGAACCTT			
pcoD	RP-pcoD-F	TATTGTCCTGCCTGCTGATG	55.0	126	This study
	pcoD-R	GATGGGTCAGATCGCTCAGT			(Chalmers et al., 2018)
tcrB	tcrB-F	CATCACGGTAGCTTTAAGGAGATTTTC	55.0	663	(Hasman et al., 2006)
	tcrB-R	ATAGAGGACTCCGCCACCATTG-			
czrC	czrC-F	TAGCCACGATCATAGTCATG	55.0	632	(Cavaco et al., 2011)
	czrC-R	ATCCTTGTTTTCCTTAGTGACTT			

[†] Probe sequences each contained a 5' FAM fluorophore and 3' black hole quencher combination for use in probe-based 5' nuclease assays; probe concentration of 100nM; primer concentration of 600nM.
[‡] Tm (°C) is the annealing temperature at which the PCR assay was performed.
[§] PCR product refers to the expected amplification product size in nucleotide base pairs (bp).

References

Aminov, R.I., Garrigues-Jeanjean, N., and Mackie, R.I. (2001). Molecular ecology of tetracycline resistance: development and validation of primers for detection of tetracycline resistance genes encoding ribosomal protection proteins. *Appl Environ Microbiol* 67(1)**,** 22-32. doi: 10.1128/AEM.67.1.22-32.2001.

Argudín, M.A., Cariou, N., Salandre, O., Le Guennec, J., Nemeghaire, S., and Butaye, P. (2013). Genotyping and antimicrobial resistance of Staphylococcus aureus isolates from diseased turkeys. *Avian pathology* 42(6)**,** 572-580.

Cavaco, L.M., Hasman, H., Aarestrup, F.M., Wagenaar, J.A., Graveland, H., Veldman, K., et al. (2011). Zinc resistance of *Staphylococcus aureus* of animal origin is strongly associated with methicillin resistance. *Veterinary microbiology* 150(3-4)**,** 344-348.

Chalmers, G., Rozas, K.M., Amachawadi, R.G., Scott, H.M., Norman, K.N., Nagaraja, T.G., et al. (2018). Distribution of the PCO gene cluster and associated genetic determinants among swine *Escherichia coli* from a controlled feeding trial. *Genes* 9(10)**,** 504.

Frahm, E., and Obst, U. (2003). Application of the fluorogenic probe technique (TaqMan PCR) to the detection of *Enterococcus* spp. and *Escherichia coli* in water samples. *J Microbiol Methods* 52(1)**,** 123-131.

Harms, G., Layton, A.C., Dionisi, H.M., Gregory, I.R., Garrett, V.M., Hawkins, S.A., et al. (2003). Real-time PCR quantification of nitrifying bacteria in a municipal wastewater treatment plant. *Environ Sci Technol* 37(2)**,** 343-351.

Hasman, H., Kempf, I., Chidaine, B., Cariolet, R., Ersbøll, A.K., Houe, H., et al. (2006). Copper resistance in *Enterococcus faecium*, mediated by the *tcr*B gene, is selected by supplementation of pig feed with copper sulfate. *Applied and Environmental Microbiology* 72(9)**,** 5784-5789.

Ng, L.K., Martin, I., Alfa, M., and Mulvey, M. (2001). Multiplex PCR for the detection of tetracycline resistant genes. *Mol Cell Probes* 15(4)**,** 209-215. doi: 10.1006/mcpr.2001.0363.

Poulsen, A.B., Skov, R., and Pallesen, L.V. (2003). Detection of methicillin resistance in coagulase-negative staphylococci and in staphylococci directly from simulated blood cultures using the EVIGENE MRSA Detection Kit. *Journal of Antimicrobial Chemotherapy* 51(2)**,** 419-421.

Sengelov, G., Halling-Sorensen, B., and Aarestrup, F.M. (2003). Susceptibility of *Escherichia coli* and *Enterococcus faecium* isolated from pigs and broiler chickens to tetracycline degradation products and distribution of tetracycline resistance determinants in *E. coli* from food animals. *Vet Microbiol* 95(1-2)**,** 91-101.

Stegger, á., Andersen, P., Kearns, A., Pichon, B., Holmes, M., Edwards, G., et al. (2012). Rapid detection, differentiation and typing of methicillin-resistant *Staphylococcus aureus* harbouring either *mec*A or the new *mec*A homologue *mec*ALGA251. *Clinical Microbiology and Infection* 18(4)**,** 395-400.

Szczepanowski, R., Linke, B., Krahn, I., Gartemann, K.H., Gutzkow, T., Eichler, W., et al. (2009). Detection of 140 clinically relevant antibiotic-resistance genes in the plasmid metagenome of wastewater treatment plant bacteria showing reduced susceptibility to selected antibiotics. *Microbiology* 155(Pt 7)**,** 2306-2319. doi: 10.1099/mic.0.028233-0.

Vikram, A., Rovira, P., Agga, G.E., Arthur, T.M., Bosilevac, J.M., Wheeler, T.L., et al. (2017). Impact of "raised without antibiotics" beef cattle production practices on occurrences of antimicrobial resistance. *Appl Environ Microbiol* 83(22)**,** e01682-01617. doi: 10.1128/AEM.01682-17.
