# Supplementary material for: Anaerobic Digestion of Tetracycline Spiked Livestock Manure and Poultry Litter Increased the Abundances of Antibiotic and Heavy Metal Resistance Genes
Source: Front Microbiol. 2020 Dec 18;11:614424. doi: 10.3389/fmicb.2020.614424 (PMC7775313; doi:10.3389/fmicb.2020.614424)
Supplement: Supplementary Table 2 — Summary of the effects of anaerobic digestion of cattle and swine manure, and poultry litter. [file Table_2.pdf]

Supplementary Table 2. Summary of the effects of anaerobic digestion of cattle and swine manure, and poultry litter.

**Measured outcome**	**Digestion**			**Overall effect**	**Reference**
	**Cattle**	**Swine**	**Poultry**		
Total bacteria	Increased	Increased	Increased	Increased	Figure 1
E. coli	Increased	No effect	Increased	Variable	Figure 1
Enterococcus spp.	No effect	Increased	Increased	Variable	Figure 1
Staphylococcus aureus	Increased	No effect	Increased	Variable	Figure 1
tet(A)	Increased	Increased	No effect	Variable	Figure 2
tet(B)	Increased	Increased	Increased	Increased	Figure 2
tet(G)	Increased	Increased	Increased	Increased	Figure 2
tet(M)	Increased	Increased	Increased	Increased	Figure 2
tet(O)	Increased	Increased	Increased	Increased	Figure 2
*tet(*Q)	Increased	Increased	Increased	Increased	Figure 2
*tet(*W)	No effect	No effect	Increased	Variable	Figure 2
erm(B)	No effect	Increased	Decreased	Variable	Figure 5
mecA	Increased	No effect	Increased	Variable	Figure 5
*mec*C	Increased	Increased	Increased	Increased	Figure 5
copB	No effect	No effect	Not detected	No effect	Text
pcoA	No effect	No effect	No effect	No effect	Figure 6
pcoD	Increased	Increased	No effect	Variable	Figure 6
tcrB	Increased	Increased	Increased	Increased	Figure 6
czrC	No effect	Decreased	Increased	Variable	Figure 6
